# Dynamic magnetic resonance imaging method based on golden-ratio cartesian sampling and compressed sensing

**DOI:** 10.1371/journal.pone.0191569

**Published:** 2018-01-30

**Authors:** Shuo Li, Yanchun Zhu, Yaoqin Xie, Song Gao

**Affiliations:** 1 Department of Biophysics, School of Basic Medical Sciences, Peking University, Beijing, China; 2 Institute of Biomedical and Health Engineering, Shenzhen Institutes of Advanced Technology, Chinese Academy of Sciences, Shenzhen, China; 3 Department of Radiology, School of Medicine, University of California, San Diego, California, United States of America; Worcester Polytechnic Institute, UNITED STATES

## Abstract

Dynamic magnetic resonance imaging (DMRI) is used to noninvasively trace the movements of organs and the process of drug delivery. The results can provide quantitative or semiquantitative pathology-related parameters, thus giving DMRI great potential for clinical applications. However, conventional DMRI techniques suffer from low temporal resolution and long scan time owing to the limitations of the *k*-space sampling scheme and image reconstruction algorithm. In this paper, we propose a novel DMRI sampling scheme based on a golden-ratio Cartesian trajectory in combination with a compressed sensing reconstruction algorithm. The results of two simulation experiments, designed according to the two major DMRI techniques, showed that the proposed method can improve the temporal resolution and shorten the scan time and provide high-quality reconstructed images.

## Introduction

Dynamic magnetic resonance imaging (DMRI) has been widely used to noninvasively trace the movement of human tissues and organs (e.g., cardiac cine MRI [1] and dynamic contrast-enhanced MRI [2]), and the process of drug delivery. The reconstructed dynamic images obtained via DMRI can be used to extract many quantitative or semiquantitative pathology-related parameters. These parameters contain considerable biological and pathophysiological information about tissues and organs during the development and occurrence of diseases. This information can be used for clinical disease research and diagnosis; therefore, DMRI has significant potential in clinical research and applications.

The DMRI technique uses three *k*-space sampling schemes: sampling based on a Cartesian trajectory [3], sampling based on a golden-angle radial trajectory [4], and hybrid sampling based on both Cartesian and radial trajectories [5]. All three schemes must repeatedly acquire all or parts of the *k*-space data. Sampling based on a Cartesian trajectory is the traditional sampling scheme and has the advantages of a simple pulse sequence design and image reconstruction method; however, this scheme suffers from low temporal resolution. The second sampling scheme stems from the golden-ratio angle sampling scheme proposed by Winkelmann in 2007 [6]. The golden-angle radial trajectory sampling scheme improves the temporal resolution of two-dimensional (2D) DMRI to a certain extent; however, three-dimensional (3D) DMRI still suffers from low temporal resolution. Furthermore, the radial trajectory introduces aliasing artifacts into the reconstructed images [7]. The hybrid sampling scheme is a combination of linear and radial trajectories; for example, in a 3D cylindrical *k*-space, Cartesian slice encoding can be used in the *k*_*z*_ direction and radial sampling can be used in the *k*_*x*_-*k*_*y*_ plane. The hybrid sampling scheme improves the temporal resolution of 3D DMRI to some extent; however, the radial trajectory part still introduces artifacts. Therefore, DMRI based on a linear sampling scheme with high temporal resolution shows significant promise for future clinical research and applications.

To realize DMRI based on a linear sampling scheme with high temporal resolution, we present two basic methods in this paper. First, because the use of the golden-ratio angle proposed by Winklemann improves the temporal resolution in radial trajectory, we introduced the golden ratio into a Cartesian trajectory. Second, compressed sensing (CS), first proposed in 2006 and widely used in many scientific fields, including fast MRI [8, 9], can be used to reconstruct relatively high-quality images from undersampled *k*-space data.

In this paper, we first discuss the basic theories of the golden-ratio-based linear sampling scheme and CS. Second, the proposed DMRI technique based on the golden-ratio Cartesian sampling scheme and CS is described. Third, computer simulation experiments conducted to compare the proposed and traditional methods are discussed. Finally, the future development of the proposed DMRI technique is outlined.

## Theory and methods

### Cartesian *k*-space sampling scheme based on the golden ratio

**Fig 1:**
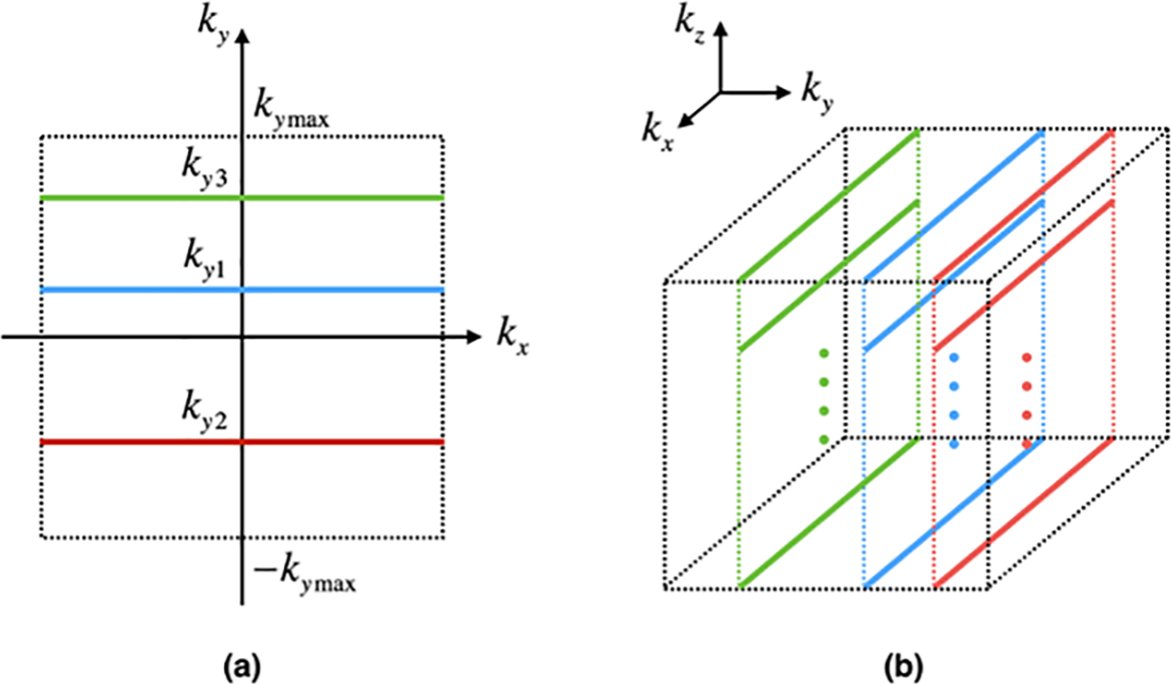
Diagrams of (a) 2D and (b) 3D Cartesian *k*-space sampling schemes based on the golden ratio.

The golden ratio is an irrational number defined as $\left(\sqrt{5}-1\right)/2$. It has been widely used in mathematics, physics, architecture, and other fields. Because of its special mathematical properties, Winkelmann proposed a *k*-space sampling scheme based on the golden-ratio angle [6]. This scheme provides approximately uniformly distributed *k*-space data within an arbitrary reconstruction window. Therefore, it offers a high degree of flexibility in choosing an appropriate window length and temporal resolution, in positioning the reconstruction window, and in combining adjacent time frames. This allows the application of sliding window reconstruction to variable window lengths and arbitrary window positioning. However, radial trajectories introduce aliasing artifacts into the reconstructed images, whereas Cartesian trajectories do not. We propose a *k*-space sampling scheme based on the golden ratio and a Cartesian trajectory. Diagrams of 2D and 3D sampling schemes are shown in Fig 1(A) and 1(B). The blue, red, and green lines in Fig 1(A) denote the first, second, and third sampling trajectories, respectively. *k*_*y*1_, *k*_*y*2_, and *k*_*y*3_ denote the corresponding coordinates in the phase-encoding direction, where *k*_*yn*_ is the coordinate of the *n*th trajectory in the phase-encoding direction and is calculated as

$$\begin{array}{c} k_{yn}=\alpha ∙k_{ymax}\\ \alpha =2mod(\gamma ∙n,1)-1 \end{array}$$ (1)

where α is the proportionality coefficient, mod(*a*,*b*) denotes the remainder of *a/b*, and γ (≈ 1.618) is the golden ratio. In Fig 1(B), *k*_*z*_ is the slice-encoding direction. All the trajectories with the same *k*_*z*_ coordinate are defined as a profile, e.g., the blue, red, and green profiles. Data in the blue profile are acquired first, followed by those in the red and green profiles. The data acquisition time of each profile is *m*_*z*_TR, where TR is the repetition time and *m*_*z*_ is the slice-encoding number.

**Fig 2:**
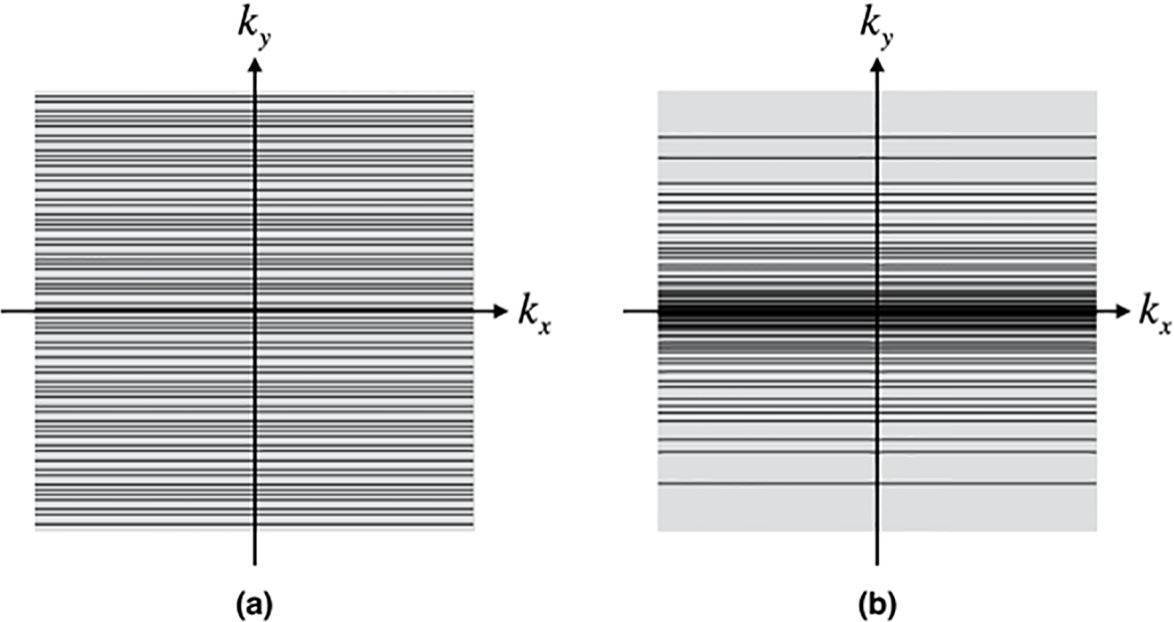
Diagrams of 64 successive 2D *k*-space sampling trajectories based on (a) Eq (1) and (b) Eq (2).

*k*_*yn*_ values, calculated by using Eq (1), are distributed uniformly along the *y*-axis. However, the low-frequency signals are concentrated near the center of the *k*-space. Given that the low-frequency signals contain the main information of the image and that information can be used to trace tissue movement, the calculation of *k*_*yn*_ can be improved as follows:

$$k_{yn}=sign(\alpha )∙(1-\sqrt{1-\alpha^{2}})∙k_{ymax}$$ (2)

where sign(*α*) denotes the sign of *α*. Fig 2(A) and 2(B) show 64 successive 2D *k*-space sampling trajectories with a spatial resolution of 256 × 256 derived using Eqs (1) and (2), respectively, where the black solid lines represent the *k*-space sampling trajectories. The improved trajectories in Fig 2(B) are denser near the *k*-space center, which could be used to reconstruct the main information of the image.

**Fig 3:**
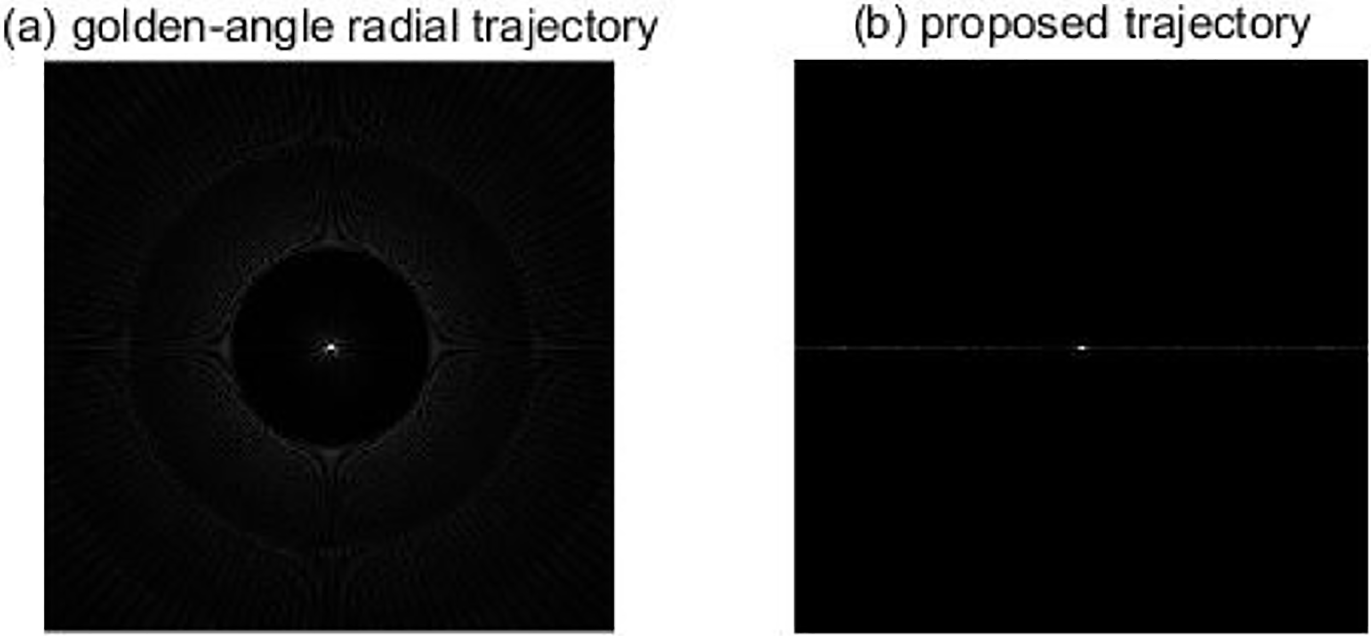
Point spread function comparison between (a) the golden-angle radial trajectory and (b) the proposed trajectory.

Fig 3 shows the point spread function of the golden-angle radial trajectory and the proposed trajectory based on Eq (2).

### DMRI reconstruction method based on CS

The CS technique can be used to precisely reconstruct the images in undersampling conditions [10]. Applying CS to MRI can significantly shorten the scanning time and provide a basis for achieving a high temporal resolution in DMRI. The fundamental concept behind DMRI image reconstruction based on CS is to solve the following optimization problem:

$$\begin{array}{c} y=Fx=F\Phi^{-1}z\\ z=\Phi x \end{array}$$ (3)

where *x* is the image, *y* is the measured *k*-space data, ***F*** is the Fourier transformation matrix, and **Φ** is a sparse matrix. Optimization of Eq (3) occurs when the vector *z* is the sparsest. Of the many algorithms that can solve Eq (3), we used a total-variation-based self-adaption algorithm [11], in which the following optimization problem must be solved:

$$\hat{x}=argmin\left\{\frac{1}{2}{\left\|y-Fx\right\|}_{2}^{2}+\lambda {\left\|\nabla x\right\|}_{1}\right\}$$ (4)

where ∇ is the difference operator matrix, ||*A*||_1_ is the L1-norm, ||*B*||_2_ is the L2-norm, arg min{*C*} is the minimization of the argument *C*, and *λ* is the regularization factor.

## Simulation experiments

**Fig 4:**
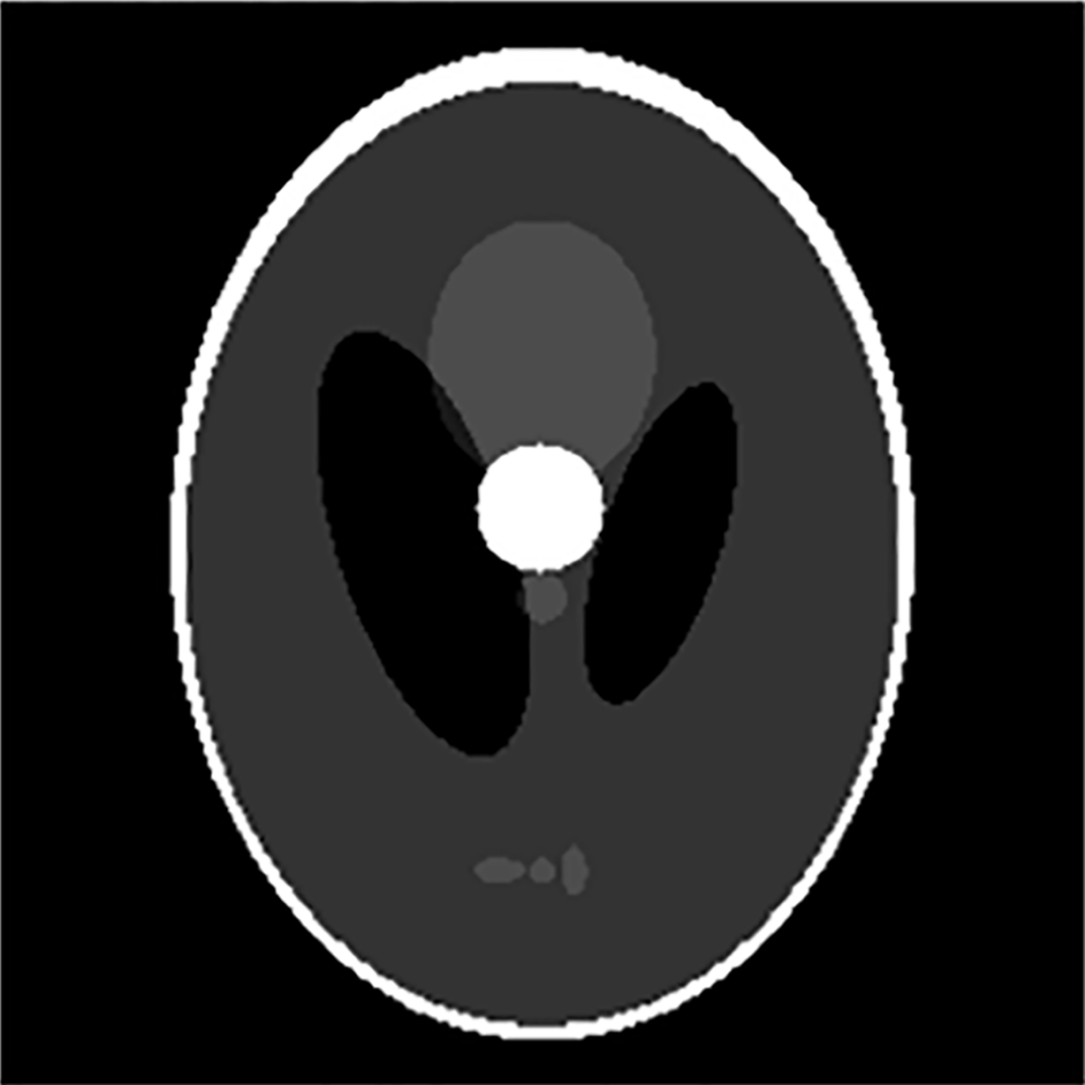
“Shepp-Logan” phantom used in the simulation.

MATLAB 7.0 (MathWorks, Natick, MA, USA) was used for 2D MRI simulation experiments in which a 2D “Modified Shepp-Logan” phantom image was used. Fig 4 shows the white ball, the movement of which simulates that of human organs or tissues. Two simulation experiments were designed to reflect the two main applications of DMRI. The periodic movement of human organs (such as cardiac motion) was imaged using three different trajectories for *k*-space data acquisition: a traditional Cartesian trajectory, a golden-angle-based radial trajectory, and the proposed sampling trajectory. The dynamic images were reconstructed using traditional Fourier transformation, regridding-based reconstruction, and CS-based reconstruction. The images reconstructed using these three methods were then compared.

### Experiment I

In experiment I, the size of the phantom image was 256 × 256 pixels, the ball was located at the center of the phantom image, and its radius varied with time as a cosine function, with a period of 640TR. The radius *r*(*t*) is expressed as

$$r(t)=15+round\left\{3cos[(\frac{t}{320}-1)\pi ]\right\},\;0\leq t<640$$ (5)

where round(*a*) indicates that the expression *a* should be rounded. *r*(*t*) is in units of pixels and has an initial value of 12 and time *t* is in units of TR.

The three image reconstruction methods used in the simulation experiments are based on retrospective reconstruction [12]. Data acquired within *N* periods are assigned to the same phase interval according to the state of motion of the phantom and then used to reconstruct the corresponding phase image. Therefore, the undersampling factor can be written as

$$\eta =\frac{N∙(t_{res}/TR)}{256}$$ (6)

where *t*_res_ is the temporal resolution and TR is the unit.

To compare the results of the three reconstruction methods, we reconstructed eight phase images that traced the states of motion of the ball, with *t*_res_ = 10 and *N* = 1–25. In addition, the mean aliasing artifact power (AP) between the eight reconstructed images and the phantom images was calculated as follows:

$$AP=\frac{\sum_{i,j}{\left||I(i,j)|-|I\prime (i,j)|\right|}^{2}}{\sum_{i,j}{\left|I(i,j)\right|}^{2}}$$ (7)

where *i* and *j* are the pixel indexes of the 2D images and I and I' are phantom images and reconstructed images, respectively.

### Experiment II

In the second experiment, the size of the phantom image was 256 × 256 pixels, the radius of the ball was 15 pixels, and the center of the ball moved linearly along the *y*-axis, where

$$y(t)=-64+round(\frac{t}{20}),\;0\leq t<2560$$ (8)

where *y*(*t*) is in pixels and *t* is in units of TR.

To compare the results of the three reconstruction methods, we reconstructed images at eight different times that traced the states of motion of the ball using 20 different *t*_res_ values in the range of 5–100. The mean AP values of the corresponding states of motion were also calculated.

### Parameters of the reconstruction method

The parameters of the regridding-based reconstruction method included the Kaiser-Bessel convolution kernel [13] with a window width of 2, *β* = 18.5547, and an oversampling ratio of 2. For the self-adapting CS reconstruction method, we set *λ* = 0.05, the adaption coefficient to 0.6, the initial search step size to 1, and the maximum number of iterations to 100, and used a Wolfe line search [14].

## Simulation results and discussion

### Tissue and organ motion simulation results

**Fig 5:**
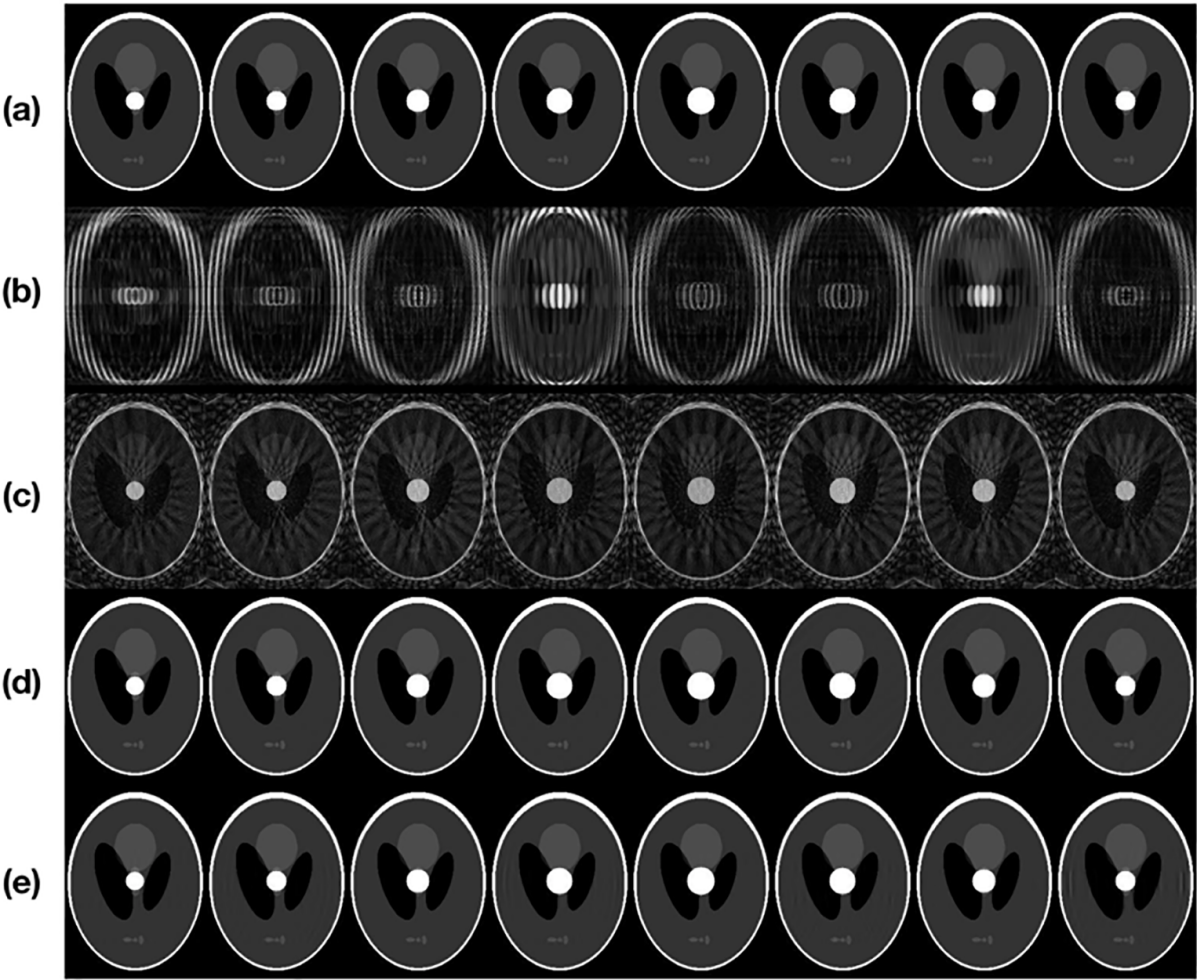
(a) phantom images, images reconstructed from (b) data from the conventional Cartesian sampling method, (c) data from golden-angle radial sampling using regridding reconstruction method, (d) data from golden-angle radial sampling using CS reconstruction method, and (e) the proposed method.

Fig 5 shows the images reconstructed using the four methods, with *t*_res_ = 10 and *N* = 7 (*η* ≈ 0.27). Fig 5(A)–5(E) show the phantom images, the images reconstructed from data acquired using the conventional Cartesian sampling method, the images reconstructed from data acquired using the golden-angle radial sampling method using the regridding reconstruction method, the images reconstructed from the data acquired using the golden-angle radial sampling method using the CS reconstruction method, and the images reconstructed using the proposed method, respectively. The images in Fig 5(B) show severe motion artifacts and the effects of undersampling. The images in Fig 5(C) have fewer motion artifacts than those in Fig 5(B); however, there are severe aliasing artifacts due to the effects of undersampling. The images for the golden-ratio angle trajectory sampling combined with the CS reconstruction method [Fig 5(D)] and the proposed method [Fig 5(E)] show better results because of the advantages of golden-ratio linear data acquisition and CS reconstruction. The results of the simulation show that both methods suppress motion artifacts and aliasing artifacts very well and yield better-quality images. However, in a clinical scan, radial trajectory sampling would suffer from the gradient delay effect caused by the MRI system, which would require additional phase correction steps [15]. The proposed method uses Cartesian *k*-space trajectory sampling, so no gradient delay-related phase correction is needed.

**Fig 6:**
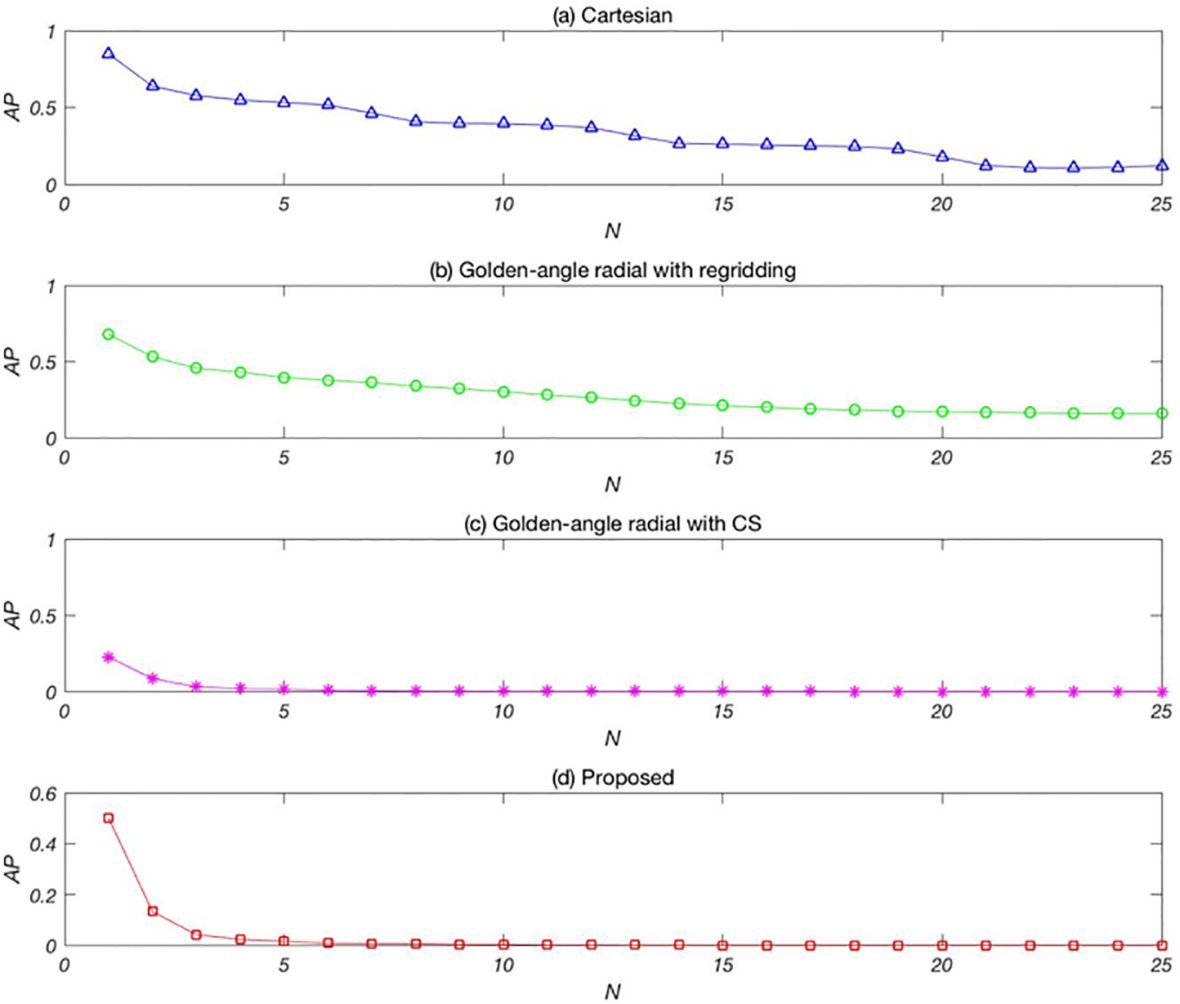
Mean AP values of reconstructed images versus *N* with a temporal resolution of 10TR.

Fig 6 shows the mean AP values of the images reconstructed from the four methods, with *t*_res_ = 10 and *N* = 1–25 (*η* ≈ 0.04–0.98): (a) the conventional Cartesian sampling method, (b) the golden-angle radial sampling method combined with the regridding image reconstruction method, (c) the golden-angle radial sampling method combined with the CS image reconstruction method, and (d) the proposed method. The AP values of the conventional Cartesian method are greater than 0.5 for *N* = 1–25, which indicates that the image quality of this method is unacceptable. The AP values of the golden-angle radial sampling method display a downward trend with increasing *N*. However, when *η* < 1, the AP value is still not ideal because the regridding image reconstruction method generally requires the acquired data to satisfy the Nyquist sampling theorem [16]. For this simulation experiment, at least 402 echoes must be acquired to meet this requirement. When *t*_res_ = 10, *N* should be >40, which is oversampling. Fig 6(C) shows that the AP value drops to nearly zero when only five periods of data (*N* = 5) are used. Under the same temporal resolution condition, the proposed method can greatly reduce the scan time. Moreover, the proposed method provides the best image quality.

### Drug delivery simulation results

**Fig 7:**
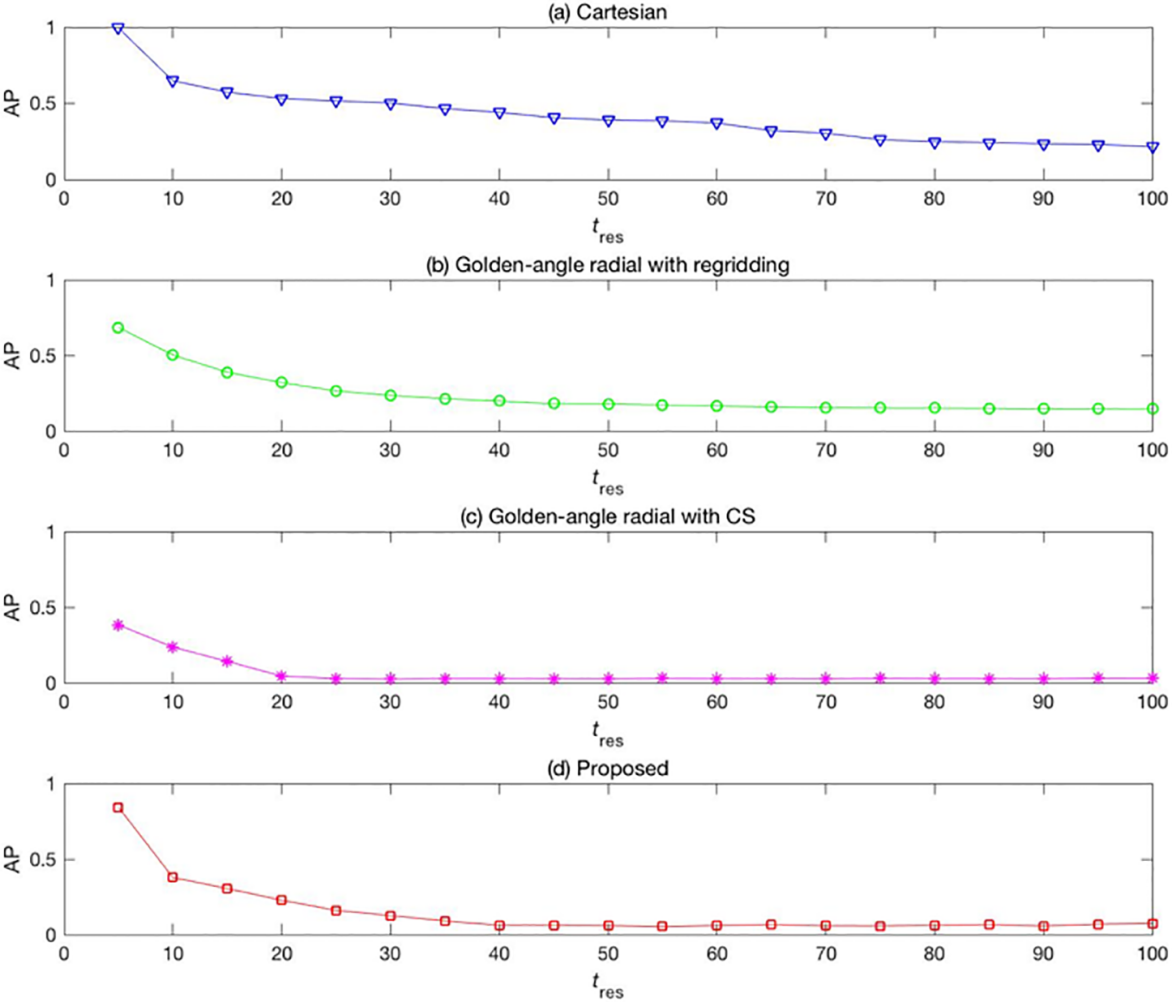
Mean AP values of the four methods with different temporal resolutions.

Fig 7 shows the mean AP values of images reconstructed from the four methods but with different temporal resolutions. The AP value is higher for a lower temporal resolution for both traditional Cartesian and golden-angle radial acquisition methods. With the traditional method, there is a tradeoff between higher image quality and lower temporal resolution. However, lower temporal resolution causes more severe motion artifacts.

**Fig 8:**
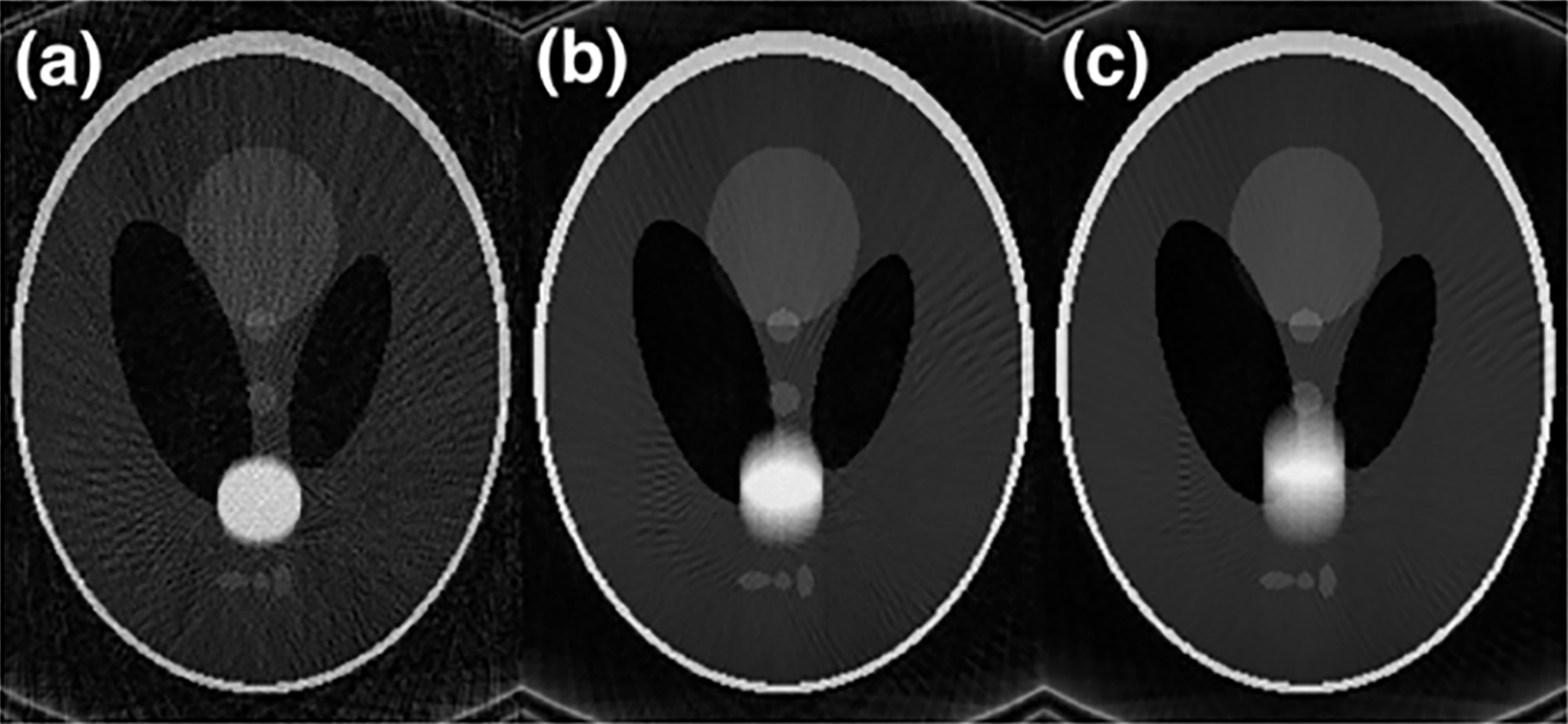
Reconstructed images from the golden-angle radial sampling method with temporal resolutions of (a) 10TR, (b) 30TR, and (c) 50TR.

Fig 8 shows that when the temporal resolution is high [Fig 8(A)], the image quality is not ideal because of undersampling. The temporal resolution of the image in Fig 8(C) is the lowest of the three images, but the state of motion of the ball is the blurriest.

The golden-angle radial sampling method combined with the CS image reconstruction method and the method proposed in this paper can provide high image quality under undersampling conditions. In addition, the image quality improves when the undersampling factor increases because the CS reconstruction algorithm allows high-quality reconstructed images from very little data. The proposed method and the golden-angle radial sampling method can provide approximately uniform *k*-space data within an arbitrary sampling window, which a conventional sampling trajectory cannot provide. Therefore, the proposed method can improve the temporal resolution.

Our results also showed that when *t*_res_ was >30 [Fig 7(C)] and 40 [Fig 7(D)], the AP values increase because when selecting more data to reconstruct a frame image, movement of the organ or tissue introduces motion artifacts in the reconstructed image, thus reducing the image quality. Therefore, it is important to select the appropriate undersampling factor and set the temporal resolution appropriately.

## Conclusion

Conventional DMRI techniques suffer from low temporal resolution and long scan time. Golden-angle radial trajectory sampling combined with a CS image reconstruction method provides better results but has a gradient delay effect, which imposes the need for additional steps to correct the phase. The proposed method improved the temporal resolution, shortened the scan time, and produced high-quality reconstructed images in the simulation experiments. In this study, we proposed and investigated DMRI that uses golden-ratio Cartesian trajectory sampling and a CS reconstruction algorithm. However, the proposed method has some problems. First, the state of motion of human organs or drug delivery in clinical scans is complex; however, the simulation experiments used in our study were based on only simple cosine and linear functions. Second, the design of the acquisition trajectories must be further optimized because linear acquisition is more sensitive to motion in the phase-encoding direction. Third, the CS reconstruction algorithm used in this study is a generic algorithm; the reconstruction algorithm needs improvement. Finally, the proposed method must be studied in more detail and validated after evaluation in a clinical situation.

## Supporting information

S1 Matlab code This is the major part of the simulation codes. (RAR)
